# A Controlled Phase 2b Trial to Assess the Efficacy and Safety of a Single Intervention of OnabotulinumtoxinA for Treating Masseter Muscle Prominence

**DOI:** 10.1093/asj/sjaf042

**Published:** 2025-03-20

**Authors:** Steven Liew, Derek Jones, Steven Dayan, Sabrina Fabi, Alexander Rivkin, Brian Biesman, Tanya Brandstetter, Grace Pan, Julia K Garcia, Elisabeth Lee, Beta Bowen, Mitchell F Brin

## Abstract

**Background:**

Masseter muscle prominence (MMP) is a benign condition characterized by a wide, square, or trapezoidal lower facial shape, which may be considered undesirable.

**Objectives:**

To evaluate onabotulinumtoxinA (onabotA) efficacy and safety for MMP treatment.

**Methods:**

In a Phase 2b study, adults with investigator- and participant-assessed bilateral Grade 4/5 MMP on the 5-grade MMP Scale (MMPS) and MMPS—Participant, respectively, were randomized 1:1:1 to receive a single intramuscular injection of onabotA 48 U, 72 U, or placebo in the masseter muscles. The primary endpoint was the proportion of patients achieving investigator-assessed MMPS Grade ≤3 at Day 90. Adverse events were monitored throughout.

**Results:**

Patients received onabotA 48 U (*n* = 53), 72 U (*n* = 46), or placebo (*n* = 46). Significantly greater proportions achieved MMPS Grade ≤3 with onabotA vs placebo (90.6%, 91.3%, and 21.7% for onabotA 48 U, 72 U, and placebo, respectively, at Day 90; *P* < .0001). Improvements in lower facial volume, width, and angle were significantly greater for onabotA vs placebo at all time points. At Day 90, the proportion of patients perceiving improvements was significantly greater with onabotA treatment vs placebo. Significantly more patients were “satisfied/very satisfied” with onabotA vs placebo through Day 180. Treatment was well tolerated; both onabotA groups had a similar incidence of treatment-emergent adverse events (TEAEs). Nasopharyngitis (onabotA, 3.9% vs placebo, 0%) and upper respiratory infection (2.9% vs 0%, respectively) were the most common TEAEs.

**Conclusions:**

One injection of onabotA 48 or 72 U was well tolerated and effective in reducing MMP severity as assessed by investigators and patients.

**Level of Evidence: 1 (Therapeutic):**

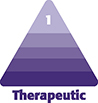

Masseter muscle prominence (MMP) is a benign clinical condition that consists of unilateral or bilateral hypertrophy of the masseter muscles, which can appear as a wide, square, or trapezoidal lower facial shape with a bulky, prominent jaw.^1,2^ MMP may occur in association with conditions, such as bruxism, temporomandibular joint disorder, occlusal and muscular imbalances, dental attrition, or chewing habits and/or diets (unilateral chewing, hard foods, or chewing gum).^1,2^ The appearance of MMP may be perceived as unattractive and have negative psychosocial impacts (eg, feeling self-conscious and reduced self-image).^3,4^ Individuals may seek MMP treatment because of aesthetic concerns, since the masseter muscle can have a substantial influence on facial shape and jawline contour.^1,2,5,6^ Concerns about looking tense or stressed may also lead an individual with MMP to seek treatment, considering a possible association of MMP with bruxism and stress and anxiety.^5^

OnabotulinumtoxinA (onabotA) is a long-acting neuromodulator that interferes with neurotransmission between nerve endings and skeletal muscle. This temporary chemical denervation is followed by a relaxation and/or decrease in muscle function that leads to a reduction in muscle bulk or volume over time.^7,8^

OnabotA has been used off-label to treat MMP for >2 decades.^7-12^ Numerous reports in the medical literature have indicated that onabotA treatment of the masseter muscles is safe and effective for improving the appearance of the lower face, but large, long-term, randomized controlled studies across diverse patient populations have yet to be conducted. In a 12 month, multicenter, double-blind, randomized, placebo-controlled, Phase 2 trial (NCT02010775), onabotA significantly reduced masseter volume and MMP severity across 4 different doses (24, 48, 72, and 96 U) with a dose-dependent trend favoring the 3 higher doses. The treatment was well tolerated, with no new safety concerns attributable to higher doses, although an impact on smile was reported in the 96 U onabotA dose group as a local effect.^13^

Here, we report the results of a Phase 2b study in which the efficacy and safety of onabotA 48 and 72 U were evaluated over 6 months in adults with bilateral Grade 4 or 5 MMP (NCT03861936).

## METHODS

### Study Design

This was a randomized, double-blind, placebo-controlled Phase 2b study to evaluate the efficacy and safety of a single intervention with onabotA 48 or 72 U for the treatment of bilateral MMP. The study was approved by an institutional review board (Advarra, Columbia, MD) before initiation at 14 study sites in the United States, and was conducted between May 2019 and July 2020 in accordance with the International Council on Harmonization guidelines for Good Clinical Practice, the principles of the Declaration of Helsinki, and the Council for International Organizations of Medical Sciences (CIOMS) Ethical Guidelines. All participants or their legally authorized representatives provided written informed consent.

### Participants and Randomization

Eligible participants were male and female adults with body mass index ≤30 kg/m^2^ who had “marked” (Grade 4) or “very marked” (Grade 5) bilateral MMP at the Day 1 visit, as determined by the investigator using the Masseter Muscle Prominence Scale (MMPS), and “pronounced” (Grade 4) or “very pronounced” (Grade 5) bilateral MMP, as determined by the participant using the Masseter Muscle Prominence Scale—Participant (MMPS-P). All participants must also have been assessed by the investigator as able to follow study instructions, and participants of childbearing potential must have been willing to minimize the risk of pregnancy during the study. Exclusion criteria were any history or condition that would contraindicate exposure to onabotA, could interfere with assessment of change in the treated area, was uncontrolled, or created an anticipated need for surgery or overnight hospitalization during the study; weakness of the masseter, pterygoid, or temporalis muscles that could interfere with normal chewing and jaw clenching; a history of or current temporomandibular joint disorder; any medical condition that could interfere with neuromuscular function; previous exposure to botulinum toxin of any serotype to the masseter area or the lower face at any time or to any part of the body 6 months prior to study initiation; or participation in another investigational drug or device study currently or within 30 days of Day 1 (Supplemental Table 1).

Participants were grouped at each investigator site based on their MMP grade (4 or 5) at baseline and randomized on Day 1 in a 1:1:1 ratio to 1 of 3 intervention arms: onabotA 48 U, onabotA 72 U, or placebo. After the randomization/treatment visit on Day 1, monthly follow-up visits occurred on Days 30, 60, 90, 120, 150, and 180. Final study exit assessments took place on Day 180.

### Treatment Administration

Each treatment consisted of a total of 6 intramuscular injections (3 injections/masseter) to the area of maximal muscle bulge using an appropriately sized needle at the discretion of the injector, as previously described.^13^ For each injection, the needle was inserted perpendicularly to the full depth of the muscle, and volume was distributed within the deeper and more superficial muscle layers. Injections were spaced ∼1 cm apart from each other and within 1 cm of the anterior border of the masseter muscle to reduce the risk of affecting neighboring anatomical structures (Supplemental Figure 1). OnabotA 48 and 72 U and placebo (0.9 mg sodium chloride) were reconstituted in preservative-free 0.9% sodium chloride. The final reconstituted volume was the same for all injections, thus facilitating investigators and patients to remain blinded throughout the study.

### Efficacy Outcome Measurements

The primary efficacy endpoint was the proportion of patients who achieved an MMPS Grade 3 or less at Day 90, per investigator assessments of MMP on the MMPS. The MMPS is a validated 5-grade clinical scale for measuring MMP severity (1 = minimal, 2 = mild, 3 = moderate, 4 = marked, 5 = very marked) that encompasses both visual and palpable examination of the masseter muscle at rest and at jaw-clench state.

Key secondary efficacy endpoints included the proportion of patients who achieved an MMPS Grade ≤3 at Day 90, per patient assessments of MMP severity on the MMPS-P, the proportion of patients who achieved a ≥2-grade improvement from baseline at Day 90 on the MMPS and the MMPS-P, the proportion of patients who achieved Participant Self-Assessment of Change (PSAC) Grade ≥2 (at least moderately improved from baseline) at Day 90, and the change from baseline to Day 90 in lower facial volume calculated from standardized images.

The MMPS-P is a validated measure that asks patients to assess the appearance of their lower face with a single score out of 5 severity grades (1 = not at all pronounced, 2 = mildly pronounced, 3 = moderately pronounced, 4 = pronounced, 5 = very pronounced) using an area-of-interest diagram showing where the masseter muscle is located (bottom half of their face from the top of their cheeks to their chin) and a 2-dimensional (2D) image derived from a 3-dimensional (3D) image of their face that was collected at the visit. A meaningful change on the MMPS-P is a 1- or 2-grade improvement as defined according to the US FDA guidance for industry for patient-reported outcomes measures using both quantitative and qualitative methods.^14^ Anchor-based quantitative methods suggested a 2-grade change is meaningful to patients, whereas qualitative participant interviews indicated a 1.4-grade change is meaningful. For the PSAC, patients were asked to refer to the area-of-interest diagram and then rate the change in appearance to the shape of their lower face by comparing their images taken before and after treatment on the following grading scale: 3 = much improved, 2 = moderately improved, 1 = minimally improved, 0 = no change, −1 = minimally worse, −2 = moderately worse, and −3 = much worse. Measurements of changes in volume of the lower face were based on 3D images captured with a VECTRA M3 3D stereophotogrammetry system (Canfield Scientific, Parsippany, NJ) and calculated using a series of predetermined anatomical landmarks placed on the baseline surface and projected mathematically to later time points.

Exploratory endpoints included the proportion of patients who reported that they were “very satisfied” or “satisfied” with MMP treatment on the Lower Facial Shape Questionnaire–Treatment Satisfaction Assessment (LFSQ-TXSAT) and the changes from baseline in lower facial width and mandibular facial angle 1C, based on 2D image projections. For the LFSQ-TXSAT, patients were asked to assess how satisfied they were with the effect of treatment on the appearance of their lower face on a 5-grade scale, where +2 = very satisfied, +1 = satisfied, 0 = neither satisfied nor dissatisfied, −1 = somewhat dissatisfied, and −2 = very dissatisfied. The mandibular facial angle 1C was defined as the internal angle generated by the intersection of the line from the earlobe attachment to the jawline/stomion line, and a vertical line parallel to the midline, for each side.

### Safety Assessments

Patients were monitored for adverse events (AEs) throughout the study. In addition, changes from baseline in vital signs, such as pulse rate, respiratory rate, and blood pressure, were assessed at all time points up to Day 180.

### Statistical Analysis

Based on previous data and an assumption of a difference of ≥34% in the responder rate between the onabotA treatments and placebo, it was determined that ∼50 patients per group would be required to provide >90% power using a 2-sided Mantel–Haenszel test, 10% dropout rate, and 5% significance level.

Efficacy analyses were conducted in the modified intent-to-treat (mITT) population, which comprised all patients who received study treatment and had ≥1 postbaseline MMPS assessment. The last-observation-carried-forward approach was used to impute missing postbaseline values. All statistical tests were 2-sided hypothesis tests performed at the 5% level of significance for main effects. For binary endpoints, *P* values for pairwise comparisons were calculated using a Cochran–Mantel–Haenszel model stratified by baseline MMPS (Grade 4 or 5).

Between-treatment comparisons of changes from baseline in lower facial volume were analyzed using analysis of covariance (ANCOVA), with treatment and investigator site as factors and baseline MMPS grade as a covariate. Between-treatment comparisons of changes from baseline in lower facial width and mandibular facial angle 1C were analyzed using ANCOVA, with treatment and investigator site as factors and baseline MMPS grade and baseline scores as covariates.

Safety assessments were conducted in all treated patients who received ≥1 administration of study intervention. For vital signs, the last nonmissing assessment before the study intervention was used as the baseline for all analyses.

## RESULTS

### Study Population

Of 227 patients screened, 150 were enrolled and randomized to receive onabotA 48 U (*n* = 53), onabotA 72 U (*n* = 49), or placebo (*n* = 48). Among the 77 patients who failed screening, the most common reasons for failure were a nonqualifying MMPS score (*n* = 26) or MMPS-P score (*n* = 23). Most patients completed the study (77.3%, 116/150), and no patients discontinued because of AEs. The most common reasons for discontinuation were “other” (8.7%, 13/150) and “lost to follow-up” (7.3%, 11/150). Notably, the COVID-19 pandemic began during the last months of the study, and 12 of the 13 patients who discontinued for “other” reasons did so for COVID-19-related issues.

In the mITT population (*n* = 145), 46 patients received placebo, 53 received onabotA 48 U, and 46 received onabotA 72 U. Most patients were female (*n* = 130 [89.7%] vs male *n* = 15 [10.3%]) and White (75.9%), with a mean age of 39.3 years (range, 18-73 years). The groups were balanced with respect to demographic and baseline clinical characteristics (Table 1); however, the placebo group had more severe baseline MMPS-P scores than the onabotA groups (47.8% of patients had very pronounced/Grade 5 MMPS-P in the placebo group vs 34.0% and 34.8% in the onabotA 48 and 72 U groups, respectively).

**Table 1:** Demographics and Baseline Clinical Characteristics (mITT Population)

	Placebo (*n* = 46)	OnabotA 48 U (*n* = 53)	OnabotA 72 U (*n* = 46)	Total (*n* = 145)
Age, years, mean (SD)	41.0 (12.1)	38.3 (10.5)	38.9 (10.6)	39.3 (11.1)
Sex, female, *n* (%)	43 (93.5)	45 (84.9)	42 (91.3)	130 (89.7)
Race, *n* (%)				
White	36 (78.3)	42 (79.2)	32 (69.6)	110 (75.9)
Asian	7 (15.2)	8 (15.1)	10 (21.7)	25 (17.2)
Black or African American	2 (4.3)	1 (1.9)	3 (6.5)	6 (4.1)
Other	1 (2.2)	2 (3.8)	1 (2.2)	4 (2.8)
Ethnicity, *n* (%)				
Hispanic or Latino	4 (8.7)	13 (24.5)	9 (19.6)	26 (17.9)
Not Hispanic or Latino	42 (91.3)	40 (75.5)	37 (80.4)	119 (82.1)
Body mass index, kg/m^2^, mean (SD)	23.9 (3.7)	24.8 (3.2)	23.5 (3.1)	24.1 (3.4)
Baseline MMPS, *n* (%)				
4 = Marked	29 (63.0)	32 (60.4)	28 (60.9)	89 (61.4)
5 = Very marked	17 (37.0)	21 (39.6)	18 (39.1)	56 (38.6)
Baseline MMPS-P, *n* (%)				
4 = Pronounced	24 (52.2)	35 (66.0)	29 (63.0)	88 (60.7)
5 = Very pronounced	22 (47.8)	18 (34.0)	16 (34.8)	56 (38.6)

mITT, modified intent-to-treat; MMPS, Masseter Muscle Prominence Scale; MMPS-P, Masseter Muscle Prominence Scale—Participant; OnabotA, onabotulinumtoxinA; SD, standard deviation.

### Investigator-Assessed MMP Severity

#### Achievement of MMPS Grade ≤3

**Figure 1:**
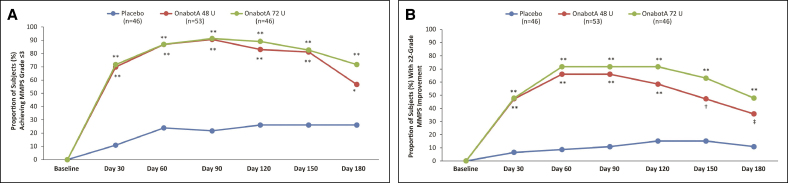
Proportion of responders achieving (A) investigator-assessed MMPS Grade ≤3 and (B) ≥2-grade change in MMPS over 180 days. MMPS, masseter muscle prominence scale; OnabotA, onabotulinumtoxinA. *P = .002 vs placebo; **P < .0001 vs placebo; ^†^*P* < .005 vs placebo; ^‡^*P* = .0042 vs placebo.

For the primary endpoint, a peak effect for a statistically significant reduction in MMP was observed on Day 90, with 90.6% and 91.3% of patients achieving an MMPS Grade ≤3 in the onabotA 48 and 72 U groups vs 21.7% in the placebo group (*P* < .0001 for each dose vs placebo; Figure 1A). Statistically significant results favoring both the onabotA 48 and 72 U groups over placebo were observed at all other time points from Day 30 to Day 180 (*P* ≤ .002; Figure 1A).

#### ≥2-Grade MMPS Improvement

For the more stringent secondary endpoint of 2-grade improvement from baseline on the MMPS, responder rates at Day 90 were statistically significantly greater in the onabotA 48 and 72 U groups (66.0% and 71.7%, respectively) compared with the placebo group (10.9%, *P* < .0001; Figure 1B). A sustained, significant response at Day 180 was observed for the onabotA 48 and 72 U doses (35.8% and 47.8%, respectively) vs placebo (10.9%; *P* < .005).

### Patient-Assessed MMP Severity

#### Achievement of MMPS-P Grade ≤3 and ≥2-Grade MMPS-P Improvement

**Figure 2:**
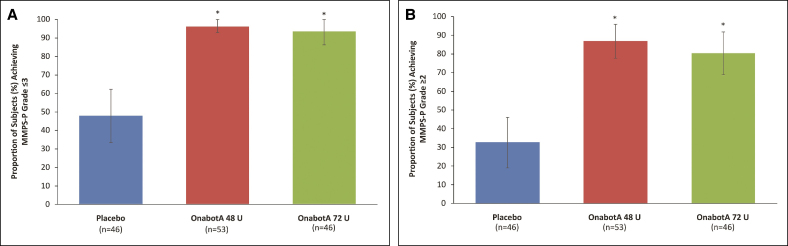
Proportion of responders achieving (A) patient-assessed MMPS-P Grade ≤3 and (B) ≥2-grade change in MMPS-P at Day 90. MMPS-P, Masseter Muscle Prominence Scale—Participant; OnabotA, onabotulinumtoxinA. *P < .0001 vs placebo.

At Day 90, the proportion of onabotA-treated patients who perceived a clinically relevant change in MMP severity (MMPS-P Grade ≤3) was significantly greater in the onabotA 48 and 72 U groups (96.2% and 93.5%, respectively) than the proportion of those who received placebo (47.8%; *P* < .0001; Figure 2A). Similarly, significantly more patients in the onabotA groups reported at least a 2-grade change in MMP severity (MMPS-P Grade ≥2; 86.8% and 80.4% in the 48 and 72 U groups, respectively) vs the placebo group on Day 90 (32.6%; *P* < .0001; Figure 2B).

#### Achievement of PSAC Grade ≥2

The patient assessment of change in MMP using the PSAC showed that the proportion of responders who perceived at least a moderate MMP improvement (≥2 grade) from baseline to Day 90 was significantly greater for both the onabotA 48 and 72 U groups compared with placebo (90.6%, 73.9.%, and 21.7%, respectively; *P* < .0001 for both comparisons).

### Objective Efficacy Measurements

#### Improvements in Lower Facial Volume, Width, and Mandibular Facial Angle

**Figure 3:**
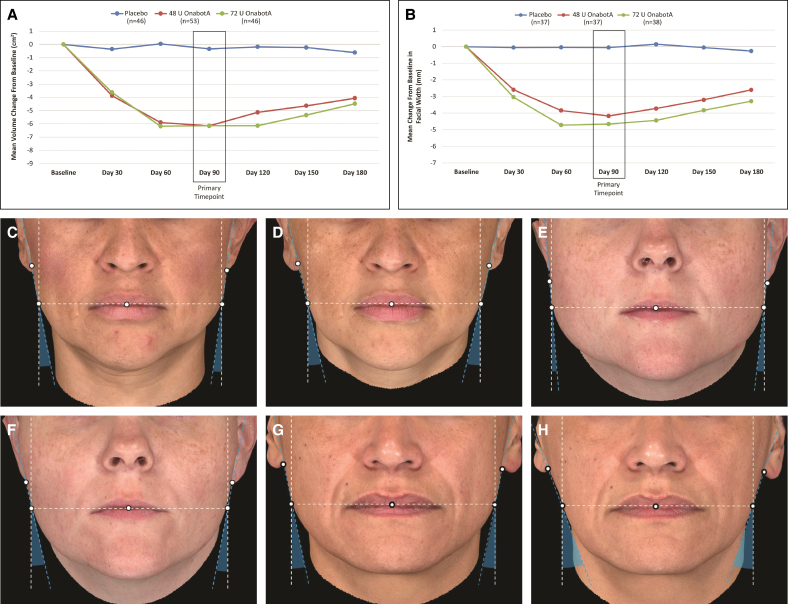
Change from baseline in (A) lower facial volume and (B) lower facial width. (C–H) Representative views of patients with MMP at baseline and after treatment with OnabotA 48 U showing increases from baseline in the mandibular facial angle 1C at Day 90. (C) A 38-year-old female at baseline and (D) 90 days after treatment. (E) A 35-year-old female at baseline and (F) 90 days after treatment. (G) A 49-year-old female at baseline and (H) 90 days after treatment. OnabotA, onabotulinumtoxinA. *P* < .0001 vs placebo at all time points.

At Day 90, lower facial volume was significantly reduced for both dose groups vs placebo (*P* < .0001; Figure 3A). The least squares mean change from baseline in lower facial volume peaked at Day 90, with a decrease of 0.33 cm^3^ in the placebo group compared with decreases of 6.15 and 6.14 cm^3^ in the onabotA 48 U and onabotA 72 U groups, respectively (*P* < .0001 vs placebo).

Significant reductions in the width of the lower face were observed at Day 90 for the onabotA 48 U (−4.17 mm) and onabotA 72 U (−4.66 mm) groups compared with placebo (−0.05 mm; *P* < .0001 vs placebo; Figure 3B). In addition, at Day 90, significant improvements from baseline in the mandibular facial angle 1C (indicating a more ovoid facial shape) were observed with both doses of onabotA (3.3° and 2.9° for 48 and 72 U, respectively) compared with placebo (0.4°; *P* < .0001 vs placebo; Figure 3C–H; see Video).

### Patient-Assessed Treatment Satisfaction

**Figure 4:**
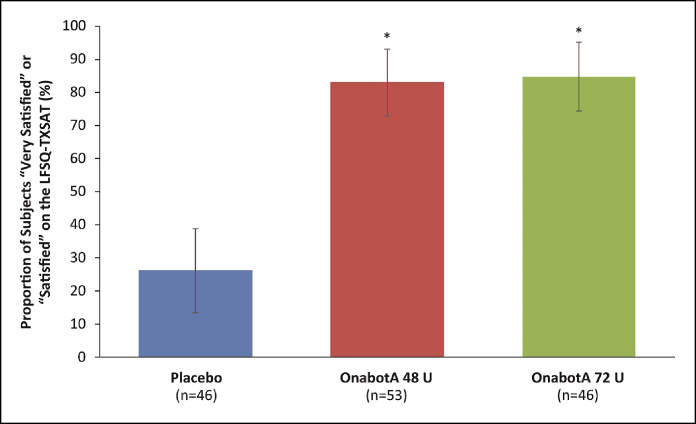
Percentage of patients who reported “very satisfied” or “satisfied” on the LFSQ-TXSAT follow-up on Day 90. *P < .0001.

In the onabotA 48 and 72 U groups, 83% and 84.8% of patients, respectively, were satisfied or very satisfied with their treatment at Day 90, compared with 26.1% in the placebo group (*P* < .0001; Figure 4). Significant differences between the onabotA groups and the placebo group were observed as early as the first measurement on Day 30 and sustained through Day 180 (*P* ≤ .0003).

### Safety Assessments

In the safety population (*n* = 150), 31.4% of onabotA-treated patients experienced treatment-emergent AEs (TEAEs), compared with 25% of placebo-treated patients (Table 2). The most common TEAEs were nasopharyngitis (4/102 [3.9%] of all onabotA-treated patients vs 0% in the placebo group) and upper respiratory infection (3/102 [2.9%] of all onabotA-treated patients vs 0% in the placebo group). No discontinuations owing to TEAEs occurred.

**Table 2:** Summary of TEAEs Occurring in at Least 3 Patients

TEAE category	Placebo (*n* = 48)	OnabotA 48 U (*n* = 53)	OnabotA 72 U (*n* = 49)	All OnabotA (*n* = 102)
Patients with at least 1 TEAE, *n* (%)	12 (25)	18 (34)	14 (28.6)	32 (31.4)
Treatment-related TEAEs, *n* (%)	0 (0.0)	5 (9.4)	6 (12.2)	11 (10.8)^a^
Injection-site pain	0 (0.0)	2 (3.8)	1 (2.0)	3 (2.9)
Muscular weakness^b^	0 (0.0)	0 (0.0)	3 (6.1)	3 (2.9)
Mastication disorder^c^	0 (0.0)	1 (1.9)	2 (4.1)	3 (2.9)
Facial paresis^d^	0 (0.0)	1 (1.9)	2 (4.1)	3 (2.9)
Treatment-emergent serious TEAEs, *n* (%)	1 (2.1)	1 (1.9)	0 (0.0)	1 (1.0)

OnabotA, onabotulinumtoxinA; TEAE, treatment-emergent adverse event.

^a^One patient experienced 2 treatment-related TEAEs.

^b^Reported as weakness at the injection site, bilateral jaw muscle weakness at injection site, or injection-site muscle weakness.

^c^Reported as difficulty chewing, increased chewing, or chewing fatigue.

^d^Reported as a weak or altered smile.

With regard to treatment-related TEAEs, the most common, each reported by 3 patients, were injection-site pain, muscular weakness (at injection site), mastication disorder (difficulty chewing or chewing fatigue), and facial paresis (reported as weak smile or altered smile). An effect on smile was reported in 1 patient in the onabotA 48 U group (1/53, 1.9%; verbatim “altered smile at injection site”), and in 2 in the onabotA 72 U group (2/49, 4.1%; verbatim “injection-related altered smile” and “weak smile”). The case reported in the onabotA 48 U group resolved spontaneously in 60 days, whereas the 2 cases in the onabotA 72 U group resolved in 79 and 101 days, respectively. Cases of mastication disorder (onabotA 48 U, 1/53 [1.9%]; onabotA 72 U, 2/49 [4.1%]) resolved within 1 month from the date of onset. All treatment-related TEAEs were mild to moderate in intensity and all resolved during the study. Two patients experienced serious TEAEs, 1 in the placebo group (fall and foot fracture) and another in the onabotA 48 U group (anxiety and suicide attempt); none were related to study drug. No AE report indicated a distant spread of the toxin.

## DISCUSSION

The results of this randomized, double-blind, placebo-controlled, Phase 2b study showed that following a single treatment, onabotA 48 and 72 U demonstrated similar statistically significant and clinically meaningful improvements from baseline in MMP compared with placebo. Improvements were observed on investigator- and patient-assessed evaluations and photography-based objective measurements. In addition, high patient satisfaction with onabotA treatment was reported throughout the study. AEs in response to treatment with onabotA 48 and 72 U were consistent with the known tolerability profile of onabotA, and no new safety findings were observed.

Investigators found significantly higher proportions of patients in the onabotA groups vs placebo achieving MMPS Grade ≤3 at Day 90. Furthermore, proportions of patients achieving a ≥2-grade change in MMPS were significantly higher (*P <* .005) at all time points through Day 180 after a single treatment with onabotA 48 and 72 U compared with placebo, with an evident dose–response trend favoring onabotA 72 vs 48 U. These data are consistent with previous findings from a Phase 2 dose-escalation study of onabotA in MMP that showed significantly higher proportions of patients achieving MMPS Grade ≤3 and ≥2-grade changes in MMPS with onabotA doses ranging from 24 to 96 U compared with placebo, and a dose-dependent trend favoring the 3 higher doses (48, 72, and 96 U).^13^

Quantitation of lower facial volume and lower facial width using 3D images has been previously used to assess the effects of onabotA on MMP, and shown to correlate well with volume reductions as measured using computed tomography and investigator-assessed severity using the MMPS tool.^13^ In this current study, measurements from 3D images showed a significant reduction from baseline in lower facial volume with onabotA treatment that peaked at Day 90 (*P* < .0001 vs placebo). These results confirm the peak effect of a clinically meaningful reduction of MMP 3 months posttreatment, consistent with published literature,^9,11,15,16^ and demonstrate similar effects to those obtained in the primary efficacy analysis. Similar volume changes from baseline were achieved in the Phase 2 dose-escalation study with onabotA 48 and 72 U.^13^ The significant reduction in lower facial width and increase in mandibular facial angle also support onabotA efficacy in MMP and the achievement of a more desirable, slimmer lower face after treatment.^4,17^

In this study, significant improvements in all efficacy assessments reported here, except for ≥2-grade improvement vs baseline on the MMPS-P in the onabotA 72 U group, were observed at Day 30, the first time point measured. These improvements steadily increased and appeared to plateau from Day 60 to Day 120, and maintained their significance through Day 180. Previous studies have reported patient-observed changes in the size of the masseter muscle as early as 2 weeks posttreatment.^18^ The maximum effect of a single treatment of onabotA in MMP has typically been observed to occur at 3 months posttreatment,^16^ with the duration of effect lasting 6 months or longer.^2,16^ In contrast, the onset of action of onabotA in the treatment of upper facial lines is within 1 to 4 days,^19^ with a peak effect observed at 14 to 30 days postinjection, and a duration of effect of 3 to 4 months.^20-28^

Patients’ perceived efficacy and satisfaction with treatment is an important goal in aesthetics. To assess the perspective of the patient, this study incorporated new and updated patient-reported outcome (PRO) assessments, including the MMPS-P, PSAC, and LFSQ-TXSAT. All PROs were developed according to US FDA PRO guidance,^29^ including qualitative interviews with participants with MMP to ensure the PROs demonstrate content validity; that is, they are relevant, well understood, and clear to participants. In addition, the MMPS-P has been psychometrically validated using data from the present study, including analysis of response distributions, test–retest reliability, agreement with the MMPS, and convergent validity. Patient-reported data were consistent with investigator-reported data, which demonstrated that patients perceived an improvement in the appearance of their lower face. In most patient assessments, efficacy appeared similar for onabotA 48 U and onabotA 72 U. PSAC findings aligned with the results obtained with the MMPS-P scale and indicated that onabotA treatment was effective in reducing MMP severity in the lower face from the patient's perspective.

In addition to these efficacy findings, onabotA was demonstrated to be a safe and well-tolerated treatment for MMP. There were no treatment-related withdrawals. AEs related to treatment with onabotA 48 and 72 U were consistent with the known tolerability profile of the toxin, and no new safety findings were observed. Safety results were comparable with those reported in previous studies of onabotA treatment for MMP, with no evidence of distant spread of the toxin. In the Phase 2 dose-escalation study of onabotA for MMP, the most frequently reported treatment-related TEAEs included commonly occurring conditions in the general population (eg, headache), events associated with the injection procedure (eg, injection-site pain), or events related to local muscle weakness that impact smiling or chewing and are consistent with the known onabotA pharmacological effects following injection into the masseter or adjacent muscles (eg, difficulty chewing or new-onset chewing fatigue, and impact on smile).^13^ The impact on smile reported in the current study occurred in 3 onabotA-treated patients across both onabotA dose groups and could be because of a transient risorius muscle dysfunction, given the proximity of this muscle to the injection site.^30,31^ Ensuring maintenance of at least 1 cm distance from the anterior border of the masseter and keeping injections as deep as possible may reduce a potential impact on the nearby risorius.

This study had some limitations. At later time points, the sample size of the study groups was smaller than expected because of high patient discontinuation due in part to COVID-19. However, despite the smaller sample size, statistical significance was still achieved. In addition, the study population was predominantly White and female; thus, the results may not be generalizable to a more heterogenous population. Further, the proportions of patients in the placebo group who achieved MMPS Grade ≤3 or a ≥2-grade improvement from baseline on the MMPS at Day 90 were relatively high; these results in the placebo group may reflect difficulties in rating a 3D volume reduction along the jawline contour with a 2D scale, as well as the unfamiliarity of clinicians and patients regarding what to expect from treatment, especially given the much smaller magnitude of the changes from baseline on objective measures (ie, lower facial volume, lower facial width, and mandibular facial angle) in the placebo group.

## CONCLUSIONS

The results of this study were consistent with the results of a prior Phase 2 dose-escalation study^13^ and suggest that treatments with onabotA 48 and 72 U are similarly well tolerated and effective in reducing MMP severity, as assessed by both clinicians and patients, for at least 6 months. OnabotA treatment significantly reduced lower facial volume and width, giving the face a more desirable ovoid shape, supported by an increased angle, and patients reported greater treatment satisfaction with both doses of onabotA compared with placebo at all time points. OnabotA provides an effective, nonsurgical, minimally invasive, well-tolerated treatment for the reduction of MMP.

## Supplemental Material

This article contains supplemental material located online at https://doi.org/10.1093/asj/sjaf042.

## Supplementary Material

sjaf042_Supplementary_Data
