# Supplementary material for: A Controlled Phase 2b Trial to Assess the Efficacy and Safety of a Single Intervention of OnabotulinumtoxinA for Treating Masseter Muscle Prominence
Source: Aesthet Surg J. 2025 Mar 20;45(10):1043–50. doi: 10.1093/asj/sjaf042 (PMC12548054; doi:10.1093/asj/sjaf042)
Supplement: sjaf042_Supplementary_Data [file sjaf042_Supplementary_Data.zip › ASJ-24-0695.R2_Masseter_Ph2_B_US_Supplemental Table 1.docx]

**Supplemental Table 1.** Eligibility Criteria

| **Inclusion Criteria** |
| --- |
| - At least 18 years of age |
| - Have “marked/very marked” (grade 4/5) bilateral masseter muscle prominence (MMP), as determined at the day 1 visit by the investigator using the Masseter Muscle Prominence Scale (MMPS) |
| - Have a “pronounced/very pronounced” (grade 4/5) MMP, as determined at the day 1 visit by the participant using the MMPS-Participant (MMPS-P) |
| - Body mass index (BMI) ≤30 kg/m^2^ using the calculation: BMI = weight (kg)/[height (m)]^2^ |
| - Female participants willing to minimize the risk of inducing pregnancy for the duration of the clinical study and follow-up period - Not a woman of childbearing potential OR - A woman of childbearing potential who agrees to follow the contraceptive guidance during the treatment and follow-up periods |
| - Able, as assessed by the investigator, and willing to follow study instructions and likely to complete all required study visits |
| **Exclusion Criteria** |
| - Any medical condition that may put the participant at increased medical risk with exposure to onabotulinumtoxinA, including diagnosed myasthenia gravis, Eaton-Lambert syndrome, amyotrophic lateral sclerosis, or any other condition that might interfere with neuromuscular function |
| - Any uncontrolled medical condition |
| - An anticipated need for surgery or overnight hospitalization during the study |
| - An anticipated need for treatment with botulinum toxin of any serotype for any indication during the study (other than study intervention) |
| - History of dental or surgical procedure for lower facial shaping or masseter muscle reduction |
| - Prior mid-facial and/or lower facial treatment with nonpermanent soft tissue fillers, synthetic implantations, autologous fat transplantation, fat-reducing injectables, and/or skin-tightening laser treatments within 6 months prior to day 1 |
| - Current or planned dental or facial procedures during the study period (eg, braces, dental implants, and reconstructive or aesthetic surgery) that could interfere with MMPS, as determined by the investigator |
| - Facial hair or scarring (eg, acne) significant enough to interfere with the 3D clinical imaging assessment |
| - Current enrollment in an investigational drug or device study or participation in such a study within 30 days of day 1 |
| - Prior exposure to botulinum toxin of any serotype to the masseter muscle or lower face at any time, or to any other part of the body within the 6 months prior to day 1 |
| - Current intraoral infection, including infection of the mouth or gums, or facial skin infection requiring medical treatment, in the opinion of the investigator |
| - History of or current temporomandibular joint dysfunction (TMJD), or presence of signs/symptoms of possible TMJD, in the opinion of the investigator |
| - Weakness of the masseter, pterygoid, or temporalis muscles due to trauma, facial nerve injury, or other condition that could interfere with normal chewing and jaw clenching, as determined by the investigator |
| - Excess lower facial fat, loose or lax skin on the lower face, or parotid gland prominence that could interfere with MMPS, as determined by the investigator |
| - Significant asymmetry of left and right sides of the face that could prevent identical MMPS grading on both sides of the face, as determined by the investigator |
| - Masseter prominence due to other etiologies (eg, parotid gland infection, parotiditis, malignancy) based upon findings from the oral examination |
