# Supplementary material for: A Controlled Phase 2b Trial to Assess the Efficacy and Safety of a Single Intervention of OnabotulinumtoxinA for Treating Masseter Muscle Prominence
Source: Aesthet Surg J. 2025 Mar 20;45(10):1043–50. doi: 10.1093/asj/sjaf042 (PMC12548054; doi:10.1093/asj/sjaf042)

**Supplemental Figure 1**. Injection site diagram for masseter muscle area showing 1 side of the face. Injection sites were targeted to the area of maximal MMP (identifiable with the mouth closed and teeth clenched) and administered with the mouth gently closed and the muscle relaxed. The first injection site was placed at the bulkiest area of the masseter muscle, followed by 2 additional injections placed so that all 3 sites were approximately 1 cm apart from each other and within 1 cm of the anterior border of the masseter muscle. MMP, masseter muscle prominence. Republished with permission under the Creative Commons Attribution (CC-BY 4.0) from Carruthers J, Liew S, Rivers JK, et al. Reduction of masseter muscle prominence after treatment with onabotulinumtoxinA: primary results from a randomized phase 2 study. *J Am Acad Dermatol.* 2024;Nov 9. Online ahead of print. doi: 10.1016/j.jaad.2024.10.064.


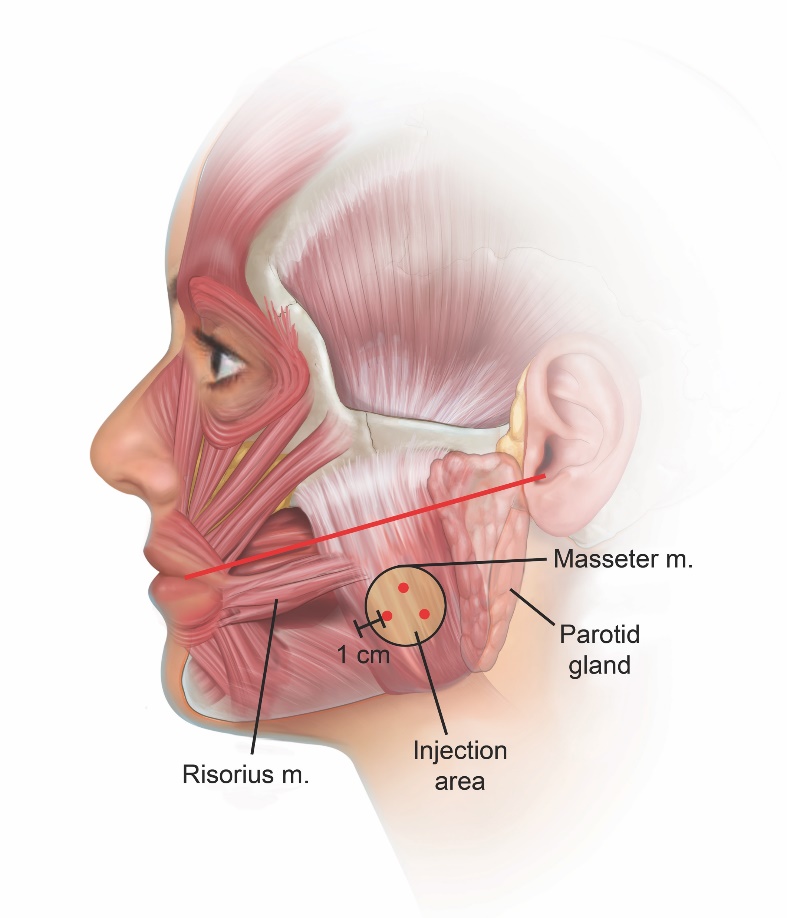

Supplement: sjaf042_Supplementary_Data [file sjaf042_Supplementary_Data.zip › Supplemental Figure 1.docx]
